# Simultaneous photon counting and charge integrating for pulse pile-up correction in paralyzable photon counting detectors

**DOI:** 10.1088/1361-6560/ace2a9

**Published:** 2023-07-19

**Authors:** Kevin Treb, Jeff Radtke, Wesley S Culberson, Ke Li

**Affiliations:** 1 Department of Medical Physics, School of Medicine and Public Health, University of Wisconsin-Madison, 1111 Highland Avenue, Madison, WI, United States of America; 2 Department of Radiology, School of Medicine and Public Health, University of Wisconsin-Madison, 600 Highland Avenue, Madison, WI, United States of America

**Keywords:** x-ray detector, x-ray imaging, photon counting CT, photon counting detector

## Abstract

***Objective*.:**

In photon counting detectors (PCDs), electric pulses induced by two or more x-ray photons can pile up and result in count losses when their temporal separation is less than the detector dead time. The correction of pulse pile-up-induced count loss is particularly difficult for paralyzable PCDs since a given value of recorded counts can correspond to two different values of true photon interactions. In contrast, charge (energy) integrating detectors work by integrating collected electric charge induced by x-rays over time and do not suffer from pile-up losses. This work introduces an inexpensive readout circuit element to the circuits of PCDs to simultaneously collect time-integrated charge to correct pile-up-induced count losses.

***Approach*.:**

Prototype electronics were constructed to collect time-integrated charges simultaneously with photon counts. A splitter was used to feed the electric signal in parallel to both a digital counter and a charge integrator. After recording PCD counts and integrating collected charge, a lookup table can be generated to map raw counts in the total- and high-energy bins and total charge to estimate pile-up-free true counts. Proof-of-concept imaging experiments were performed with a CdTe-based PCD array to test this method.

***Main results*.:**

The proposed electronics successfully recorded photon counts and time-integrated charge simultaneously, and whereas photon counts exhibited paralyzable pulse pile-up, time-integrated charge using the same electric signal as the counts measurement was linear with x-ray flux. With the proposed correction, paralyzable PCD counts became linear with input flux for both total- and high-energy bins. At high flux levels, uncorrected post-log measurements of PMMA objects severely overestimated radiological path lengths for both energy bins. After the proposed correction, the non-monotonic measurements again became linear with flux and accurately represented the true radiological path lengths. No impact on the spatial resolution was observed after the proposed correction in images of a line-pair test pattern.

***Significance*.:**

Time-integrated charge can be used to correct for pulse pile-up in paralyzable PCDs where analytical solutions may be difficult to use, and integrated charge can be collected simultaneously with counts using inexpensive electronics.

## 1. Introduction

Semiconductor-based photon counting detectors (PCDs) are rapidly emerging for medical x-ray and CT imaging applications due to advances in semiconductor sensors, allowing for faster charge collection and short afterglow. However, even with recent technological advances, pulse pile-up can still occur when electric pulses induced by two or more x-ray photons overlap, e.g. when they have a temporal separation of less than the detector dead time (Muller 1973). Pulse pile-up can result in a reduction in recorded counts and distortion of the x-ray energy spectrum. Consequently, pulse pile-up degrades the quantitative accuracy of both nonspectral and spectral PCD images (Taguchi *et al* 2010, 2011, Wang *et al* 2011, Roessl *et al* 2016, Wang *et al* 2019). The pile-up effect also impacts PCD image quality by changing the statistical distribution of the recorded counts and introducing peculiar noise texture and edge-enhancing effects (Ji *et al* 2021) that may confound or mislead physicians’ interpretation of images.

The severity of the pile-up effect is fundamentally determined by the photon flux (counts per second per area) and the PCD dead time. For example, in clinical CT imaging, a typical flux level (Taguchi *et al* 2009, Szczykutowicz *et al* 2022) is around 10^8^ counts per second per square mm (cps mm^−2^) at the detector surface, which requires the dead time of a PCD with 0.3mm pixels to be <12 ns to keep the count loss below 10%. For lower-end PCDs, the dead time can be 63 ns (single-pixel mode) or 1400 ns (anti-coincidence mode) (Ji *et al* 2019), which leads to over 75% count losses at 10^8^ cps mm^−2^ even for smaller 0.1 mm pixels.

Several hardware-based methods have been used to reduce the severity of pulse pile-up: the first and most obvious choice is to shorten the duration of the electronic pulses induced by x-ray interactions in the detector by using pulse shaping circuitry with shorter time constants. However, there exists a physical lower limit to the achievable pulse duration, which is the collection time for the electron-hole pairs in a semiconductor sensor for direct conversion PCDs. Reducing this charge collection time requires semiconductor sensors that are pristine by modern manufacturing standards, which may be costly to manufacture; even so, it may take an average of ~25 ns to finish collecting the signal from all of the electrons and holes from a 60 keV x-ray in a 1.6 mm thick CdTe sensor. If the time constant for the electronic processing of the collected electric signal, which is closely related to the dead time, is less than the physical charge collection time, the height of the resulting electric pulse can be distorted, resulting in a distorted measured energy of that x-ray photon. Further, reducing the pulse shaping time can decrease the signal-to-noise ratio of the electric pulse height (Knoll 2010). A second strategy is to reduce the detector pixel area so that for a given x-ray flux the counts per second per pixel are reduced. Although pulse pile-up may be reduced this way, there is a trade-off between pixel size and cross-talk of signal between pixels (Taguchi and Iwanczyk 2013, Taguchi 2017, Taguchi *et al* 2020, Ji *et al* 2021) such as charge-sharing, K-fluorescence, and Compton scatter, all of which distort the recorded energy spectrum. The effects of cross-talk between detector pixels can be compensated for through the use of charge-summing circuitry, but such circuitry can increase the detector dead time (Ji *et al* 2019, 2021) and further worsen pulse pile-up effects. Lastly, for CT imaging a bowtie filter and fluence-field modulation may be implemented to reduce the x-ray flux incident on the detector for less attenuating regions (Szczykutowicz and Mistretta 2013a, 2013b, Hsieh and Pelc 2013). However, fluence-field modulation has not yet been implemented in real imaging systems, and x-ray imaging systems other than MDCT typically do not have bowtie filters. Even with fluence modulation, the count rate on the detector surface can still be as large as 5 × 10^7^ cps mm^−2^, which still requires the dead time of a PCD with 0.3 mm pixels to be less than the average charge collection time of 25 ns to keep the count loss below 10%.

When situations arise where pulse pile-up cannot be avoided, several strategies have been developed to correct the pulse pile-up. Hardware-based pile-up rejectors have been explored to reduce the spectral distortion due to pile-up. However, the efficacy of such rejectors could be limited (e.g. 50%) (Wielopolski and Gardner 1976, Barradas and Reis 2006, Knoll 2010, Taguchi *et al* 2010). One such technique is to use two electronic pulse processing chains; a conventional one that processed pulses as normal, and a faster one that differs through its use of a fast pulse shaping time, and is only used to determine if a recorded pulse in the normal processing chain is corrupted by pile-up. Despite this clever idea of having two processing chains in parallel where one is used to correct for the other, the efficacy is still limited by the speed of the fast pulse processing chain, which is again limited by the physical collection time of electron-hole pairs in the semiconductor sensor; this is because the fast processing chain is still operated as a photon counter. Pile-up rejectors also technically do not correct for pile-up-induced counts losses, and simply remove the pile-up-contaminated information so that it cannot impact the recorded energy spectrum: these pile-up-contaminated counts are lost, which may reduce radiation dose efficiency.

In addition to hardware-based methods, several software-based count loss correction methods have been proposed. These likelihood methods rely on analytical pulse pile-up models (Barradas and Reis 2006, Frey *et al* 2007, Taguchi *et al* 2010, 2011, Wang *et al* 2011, Xu *et al* 2013, Liu *et al* 2016, Roessl *et al* 2016, Grönberg *et al* 2018, Yang *et al* 2021) which may be inaccurate, especially for modeling detector-specific properties relating to e.g. pulse shaping, energy responses, energy threshold comparators, dead time measurements, etc. Further, these models have only been evaluated in simulations where there is access to ground truth information, which cannot always be measured in real-world systems. The models can also be nonlinear and shift-variant (Taguchi *et al* 2022), and so model accuracy at unmeasured conditions is unknown. Lastly, pile-up modeling is made more complicated by energy binning in the PCD (Wang *et al* 2011) since the number of counts in each bin has its own statistical properties.

**Figure 1.**
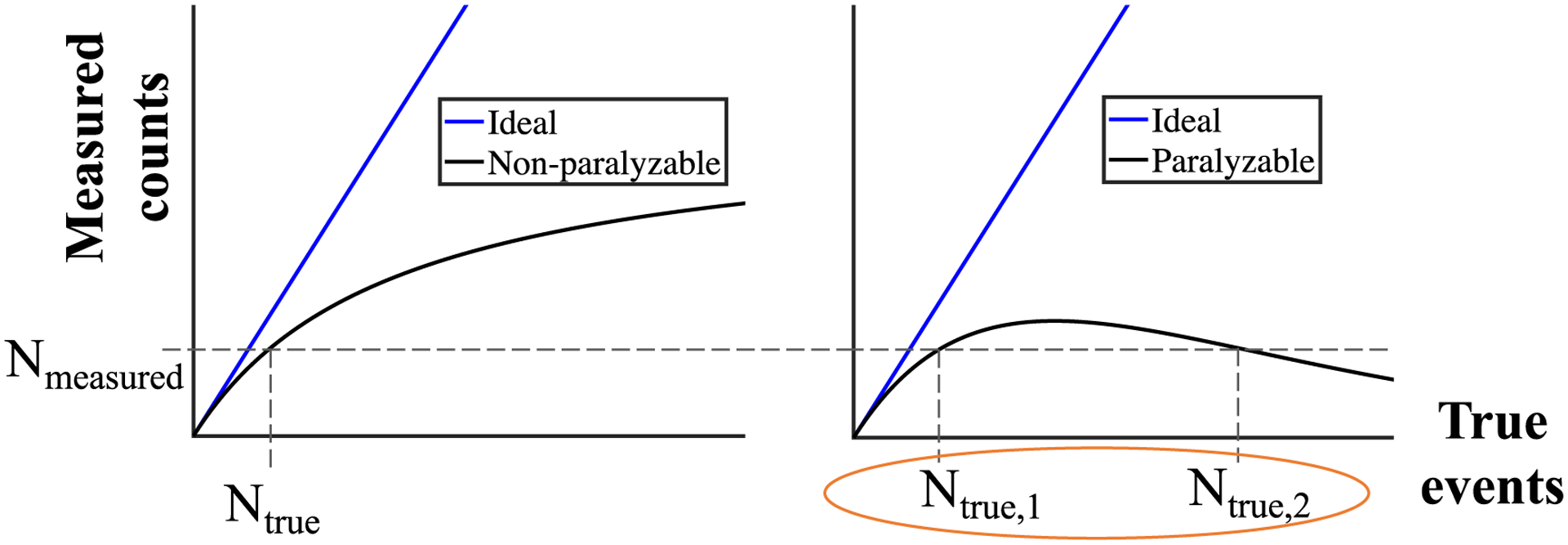
Illustrations of pulse pile-up-induced count loss for non-paralyzable and paralyzable PCD models. In paralyzable PCDs, two different true input photon numbers, *N*_true,1_ and *N*_true,2_, can generate the same *N*_measured_ value.

Despite the drawbacks, analytical pulse pile-up corrections have found reasonable success in non-paralyzable and semi-non-paralyzable (Xu *et al* 2013, Liu *et al* 2016, Grönberg *et al* 2018) models of photon counting, which despite deviations from the ideal linear relationship between measured counts *N*_measured_ and true x-ray interaction events *N*_true_, is still monotonic, and still maintains a one-to-one correspondence between measurements and ground truth no matter how high of an x-ray flux is used (figure 1). However, PCDs can exhibit paralyzable behavior where one measurement of recorded counts can correspond to two values of true x-ray interactions: Therefore, a ‘degeneracy’ challenge remains to be addressed as demonstrated in figure 1, where one *N*_measured_ may correspond to two different values for *N*_true_. For these paralyzable PCDs, previously developed analytical solutions to correct for pulse pile-up-induced count losses may be challenging without any additional information due to this degeneracy problem.

**Figure 2.**
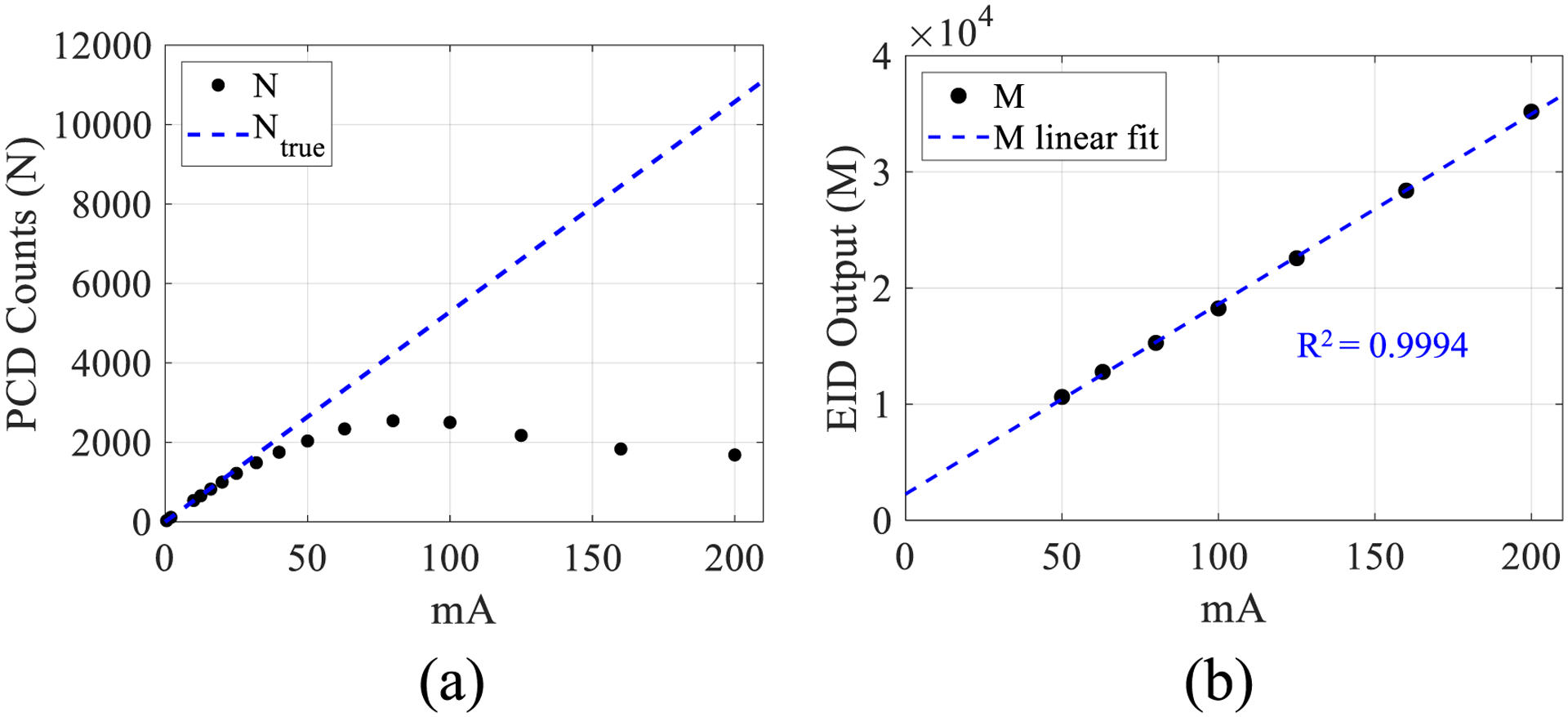
Plots of experimentally measured detector outputs versus x-ray tube current (mA) for (a) a PCD and (b) n charge integrating detector.

To address this challenge, let’s review the following facts about conventional charge (energy) integrating detectors: for a given x-ray spectrum, the charge integrating response is linear with respect to the flux without suffering from pile-up losses at high flux levels (figure 2(b)): therefore, e.g. in energy integrating detectors (EIDs) used for medical x-ray imaging, whether the arrivals of two x-ray photons are close in time does not impact the detector outputs (as long as the number of coincident events is not so great as to create an accumulation of space charge, resulting in a reduction in electric field strength, a decrease in drift velocity, and an increase in recombination losses). Further, energy integration (i.e. charge integration) is much cheaper to implement compared with pulse-mode detection (i.e. photon counting). It is also essential to recognize that the energy (charge) integration mode suffers from electronic noise contamination due to the lack of a low signal rejection mechanism. Luckily, at high flux levels that can induce pile-up in PCDs, the influence of electronic noise is usually negligible.

Inspired by the above characteristics of charge integrating detectors, we developed a method that leverages the linear response of the charge integration detection mode to correct pile-up-induced count losses in PCDs with an inexpensive upgrade of the existing photon counting circuitry to record the integrated charge in parallel. Rather than both the photon counting and the charge integrating data being used for image formation, e.g. using counts for the low flux regime and integrated charge for the high flux regime (Kraft *et al* 2007), the integrated charge is used solely for correcting the recorded photon counts, and then the corrected counts are used for image formation. This work reports experimental results generated from a prototype device, in which the analog outputs of a CdTe PCD shaping amplifier were fed to both a digital counter and a charge integrator. The outputs of the charge integrator were used to linearize the outputs of the digital counter. This work also presents imaging results from a hybrid experiment, in which a dual-energy bin PCD was used jointly with a medical x-ray EID to emulate an imager with the proposed counter + charge integrator scheme. The hybrid experiment provides the first proof of concept that the proposed method can correct pile-up-induced count losses for both energy bins of the PCD.

## 2. Materials and methods

### 2.1. Principle of the proposed correction method

In PCD x-ray and CT imaging, the Radon transform of the x-ray linear attenuation coefficient *μ*, namely $\int \mu (\vec{x})\text{d}l$, can be estimated from recorded detector counts, *N*, via the following normalization and logarithmic transform:

(1) $$\int \mu (\vec{x})\text{d}l\approx \text{ln}\frac{{\bar{N}}_{0}}{N}.$$

Because the left-hand side of equation (1) is independent of x-ray flux or mA, an important assumption of equation (1) is that $\frac{{\bar{N}}_{0}}{N}$ is independent of flux (mA). For the term ${\bar{N}}_{0}$ (expected detector counts recorded in an air scan), clinical x-ray and CT systems may require its measurement to be done for each spectrum at a relatively low mA level, mA_ref_, below the pile-up regime. For an arbitrarily high mA level, its ${\bar{N}}_{0}$ can be estimated via

(2) $${\bar{N}}_{0}(\text{mA})=\frac{\text{mA}}{{\text{mA}}_{ref}}{\bar{N}}_{0}\left({\text{mA}}_{ref}\right).$$

Therefore, pile-up-induced nonlinearity and count loss can be avoided for term ${\bar{N}}_{0}$. The real troublemaker is the term *N* (counts measured with the image object). For a paralyzable PCD (such as the experimental PCD used in this work), *N* is related to the true photon events, *N*_true_, by

(3) $$N=N_{\text{true }}\exp\left(-\frac{N_{\text{true }}}{T}\tau \right).$$

Here *τ* denotes the PCD dead time, *T* denotes the measurement time window of each detector frame, and *N*_true_/*T* is the rate of true photon events. As suggested by the experimental data in figure 2(a), *N* is underestimated at high flux/mA levels, which leads to the overestimation of ∫*μ*d*l*. Even if a *N* − *N*_true_ curve is pre-calibrated for each PCD pixel, *N*_true_ still can’t be reliably solved from *N* since one *N* value can correspond to two possible *N*_true_ values in a paralyzable PCDs (figure 1).

Unlike PCDs, the outputs of charge (energy) integrating detectors (denoted as *M*) are linearly related to *N*_true_ over a much wider range of *N*_true_ for a given spectrum. That is one of the major reasons for the success of EIDs in medical x-ray imaging in the past 50 years. For both EIDs and PCDs, their signal formations all rely on converting x-ray energy into electric charge. In charge integrating devices such as medical x-ray EIDs, this charge is accumulated during a period of *T* before it is amplified, digitized, and transferred off the detector. In PCDs, the collected charge induced by each photon event is compared with a threshold to trigger a digital count. However, this does not mean the collected charge cannot be integrated over time in a PCD. For example, some popular PCDs such as Medipix3RX and the Xcounter PCD used in this work are already equipped with an analog device to sum charge induced at each 2 × 2 pixel block for anti-coincidence correction (Ballabriga *et al* 2013). This is freely-available information without requiring a separate integration-mode acquisition. A local capacitor can be used to store all charge collected during *T* before passing the charge to an amplifier and analog-to-digital converter (ADC) to get *M*. Based on the linear, one-to-one correspondence between *M* and *N*_true_, a lookup table (LUT) that takes *N* and *M* as inputs and *N*_true_ as output can be established via a calibration process: Separate LUTs can be generated for each detector pixel of a PCD. Circuit elements to measure *M* can be installed for every detector pixel so that each pixel has its own values of counts and integrated charge. Alternatively, since some commercial PCDs already have charge summing devices for 2 × 2 pixel blocks, these already existing circuit elements can be utilized to measure *M* for a block of pixels, so that each pixel has its own value of counts, but has a value of integrated charge that is shared with its neighboring pixels. The 2 × 2 pixel block scheme is an example instead of the only scheme. It may not be optimal compared to installing charge summing circuitry for each individual pixel, but also can effectively reduce the added circuit cost and the amount of information that needs to be acquired for a given detector array.

### 2.2. Proof-of-concept experiments

#### 2.2.1. Electronics prototype

**Figure 3.**
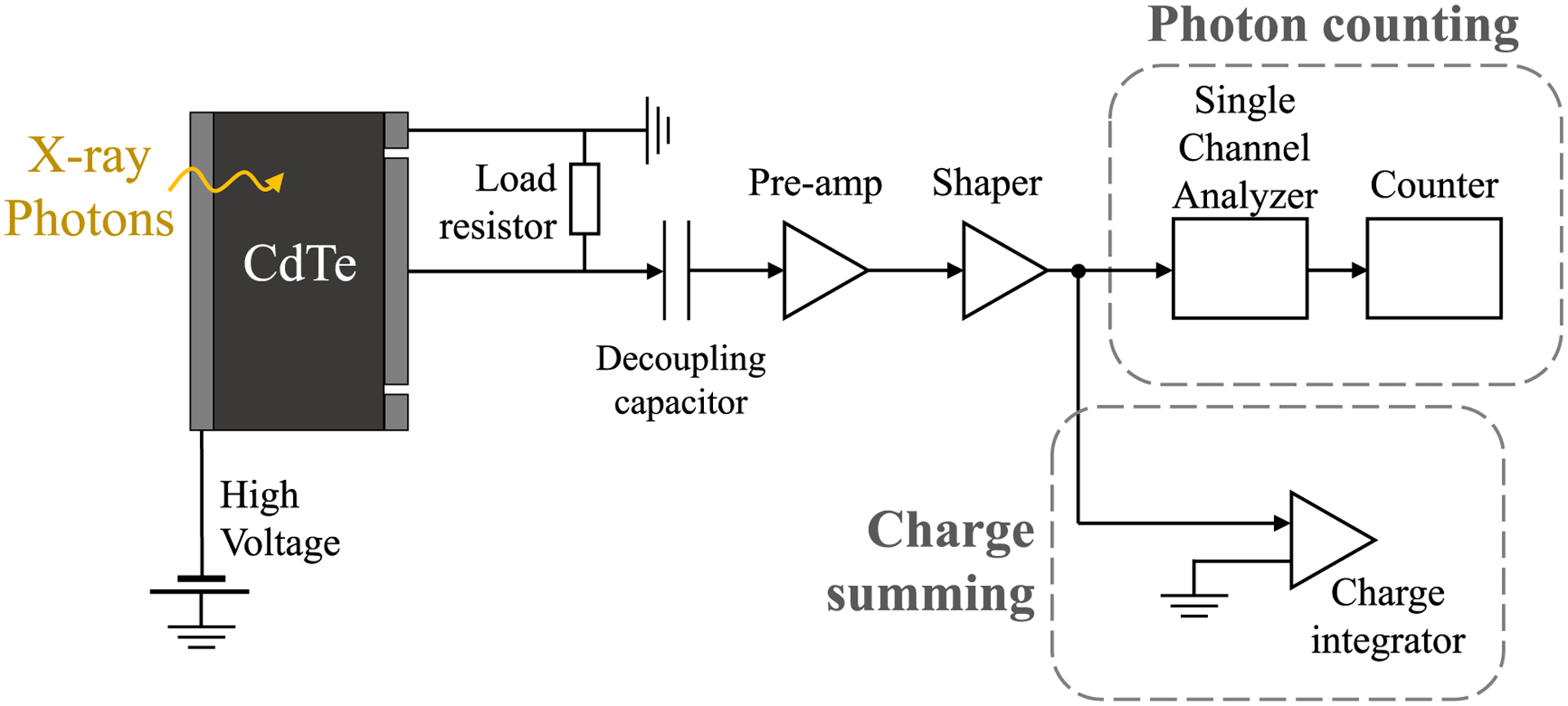
Diagram of the prototype electronics and PCD setup with photon counting and charge summing modules connected in parallel for simultaneous collection of counts and charge.

Since the proposed pile-up correction requires both the recorded photon counts and the time-integrated charge to be collected for a given x-ray exposure, prototype electronics were designed and assembled as a proof-of-concept for simultaneously acquiring counts and charges for the same imaging frame. A diagram of the prototype electronic setup is given in figure 3: for this setup, a single-element CdTe-based Schottky diode with a 4 mm × 4 mm active area was used as the x-ray detector; a reverse bias of 600 V was applied to the diode. A 0.01 *μ*F decoupling capacitor was used to isolate the bias across the diode from the rest of the electronics. The electric signal from the diode was sent through a junction field effect transistor (JFET)-based feedback charge amplifier (Radeka 1988) and a shaping amplifier (451 Spectroscopy Amplifier, Ortec) with a time constant set to 1 *μ*s for unipolar pulse shaping and a gain of 5. The shaped pulses were then sent to the counting and summing circuitry, which were connected in parallel with each other and separated by a passive splitter. The charge integrator was a Standard Imaging MAX 4000 Electrometer, which is a current amplifier with high input impedance ≫1 MΩ; the current is digitally integrated with respect to time to yield a charge reading. The photon counting module starts with a single-channel analyzer (SCA) (551 Timing Single-Channel Analyzer, Ortec) which performs pulse-height analysis: the SCA was operated in window mode and with a pulse resolving time of 800 ns. The SCA is followed by a dual counter/timer (2071A, Canberra) to count the number of digital pulses. Because the electrometer input is high impedance and the SCA thresholds are voltage-dependent, charge sharing between the electrometer and SCA inputs is not expected, and no charge sharing between the counting and summing circuitry was observed in experiments with this setup.

**Figure 4.**
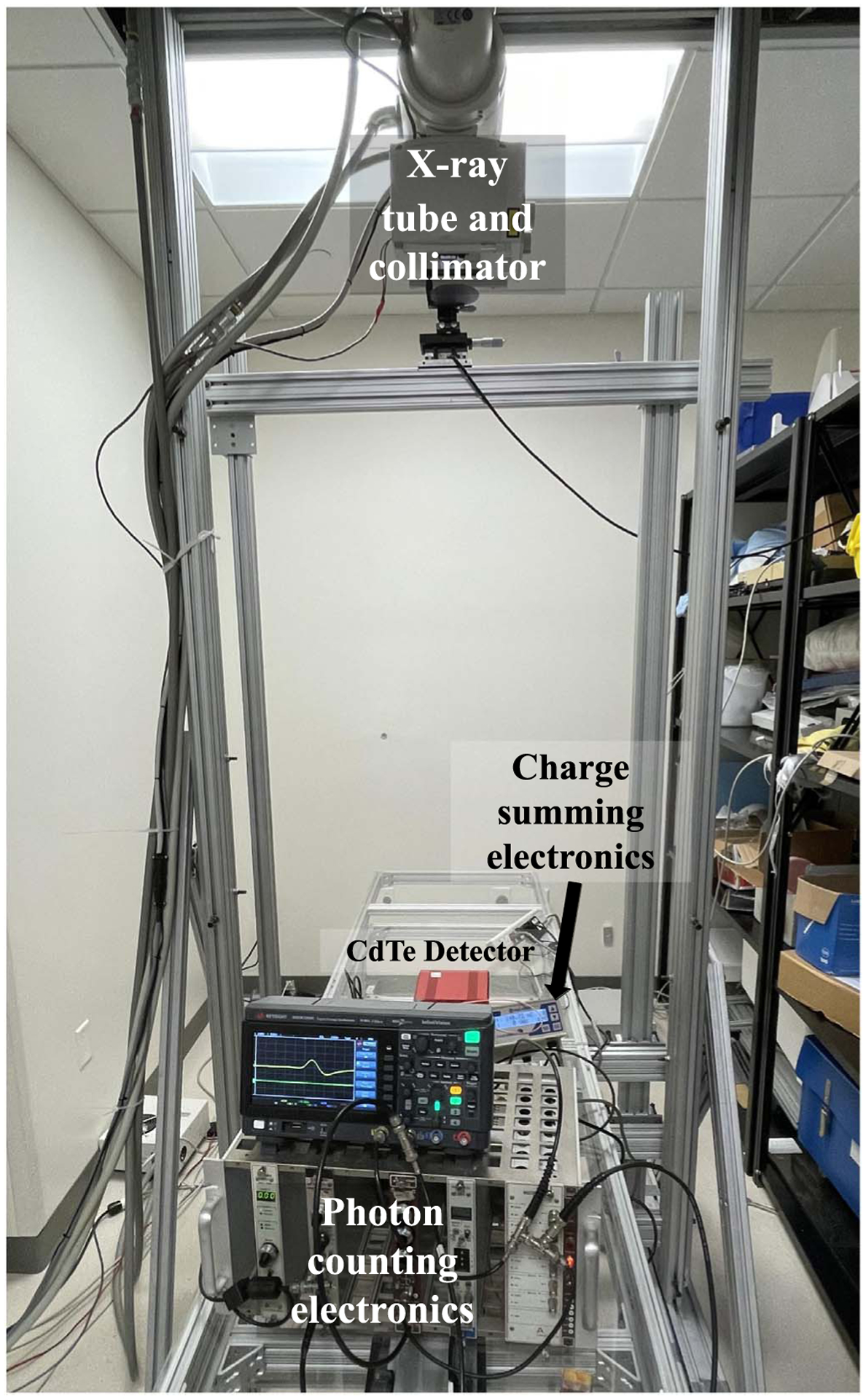
Experimental setup to test the proposed circuit design with photon counting and charge summing electronics in parallel.

**Figure 5.**
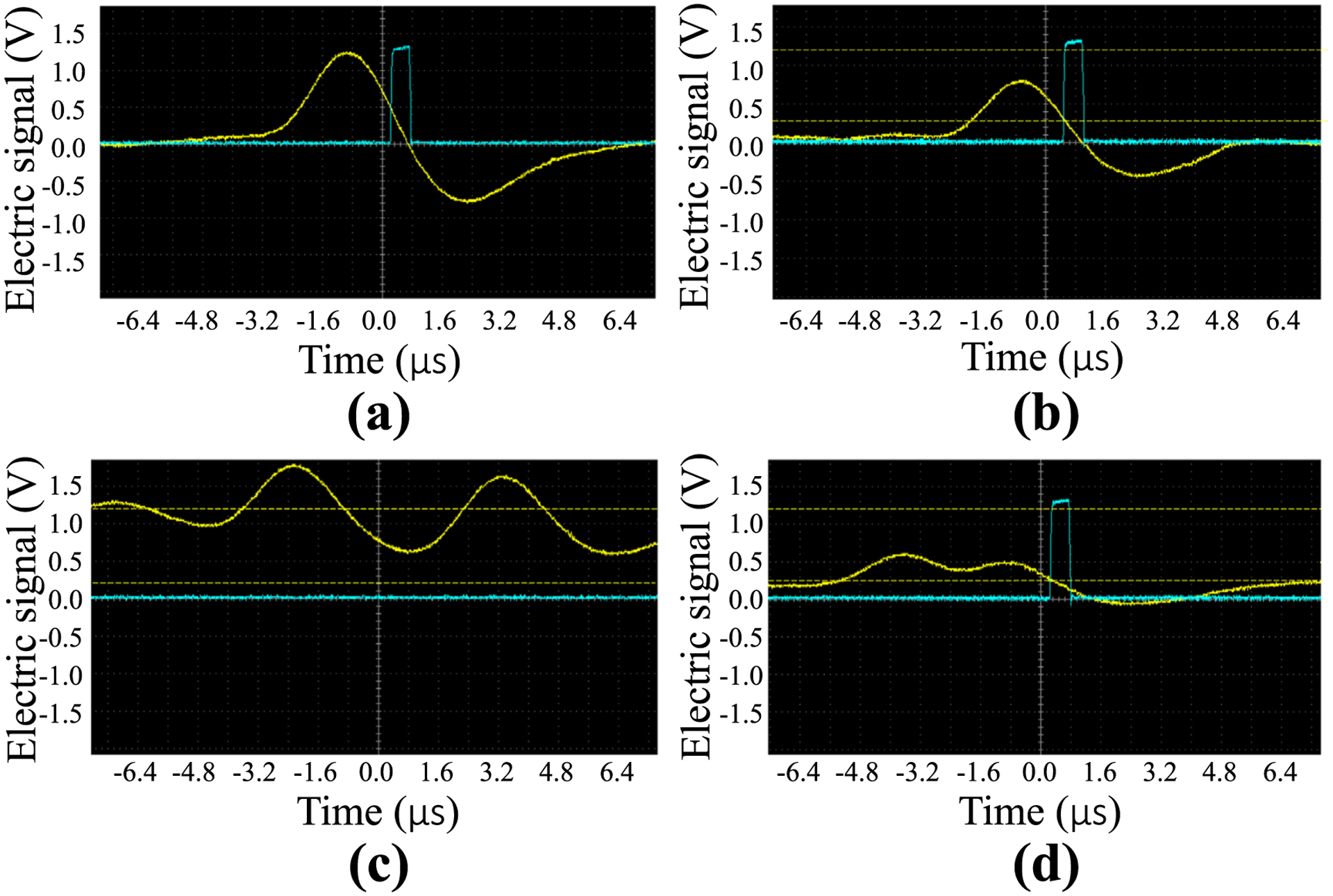
Oscillograms showing the outputs of the shaping amplifier (yellow) and the SCA (blue) for (a) an isolated peak from irradiation with an Am-241 source used to calibrate the upper SCA threshold, (b) an isolated peak from the 40 kVp beam with SCA thresholds indicated as horizontal dashed yellow lines, (c) two pulses with pile-up exceeding the upper threshold with no SCA output, and (d) two pulses with pile-up that is between the upper and lower thresholds with only one SCA output.

To test the ability of the proposed electronics to simultaneously record values of pile-up-free time-integrated charge at the same time as pile-up-corrupted photon counts, the CdTe diode detector was irradiated by x-rays from a rotating tungsten anode angiographic tube (G-1592 with B-180H housing, Varian Medical Systems, USA) shown in figure 4. Total counts and time-integrated collected charges were recorded for the same acquisition and were repeatedly measured three times at each mA level to generate error bars. The x-ray tube is powered by an 80 kW high-frequency generator (Indico 100, CPI Inc., Canada). The tube was operated at 40 kVp to keep the voltage induced by the maximum x-ray energy well below the upper threshold setting of the SCA; the primary 59.5 keV gamma rays from an Am-241 source corresponded to a pulse height of 1.2 V measured using a Hantek DSO5072P oscilloscope as shown in figure 5(a), and was used to calibrate the energy of the upper SCA threshold. With the upper energy threshold set to 1.2 V, the lower threshold was set to approximately 0.2 V to be just above the electronic noise level. The tube current for the 40 kVp x-ray beam was adjusted from 0.5 to 4 mA to obtain different pile-up levels at the detector surface: an oscillogram of a representative pulse from the 40 kVp beam is shown in figure 5(b), with horizontal dashed lines indicating the SCA thresholds. From this, it can be seen that the SCA output only begins when the electronic pulse from the amplifier crosses the lower threshold after peaking in between the lower and upper thresholds. Figure 5(c) shows how two pulses can pile up and exceed the upper threshold of the SCA while remaining above the lower threshold, resulting in no recorded pulse from the SCA; this may be a mechanism that contributes more to paralyzable behavior of pile-up. Figure 5(d) shows how two pulses can pile up and not exceed the upper threshold, but can have a trough between them that does not go below the lower threshold, resulting in only one recorded pulse from the SCA; this may be a mechanism that contributes more to non-paralyzable behavior of pile-up. Therefore, this experimental setup may have a gradual transition between non-paralyzable and paralyzable behavior. The dead time of this setup was measured using the paralyzable model to be 4.7 *μ*s; from oscillograms, the full-width at half maximum (FWHM) of the positive lobe of the output pulse from the shaping amplifier was measured to be 1.9 *μ*s and the FWHM of the negative lobe was 2.5 *μ*s.

#### 2.2.2. Pile-up correction experiments

To experimentally demonstrate the feasibility of the proposed count correction method for imaging with a PCD pixel array, an experimental x-ray imaging benchtop system was used: the system is equipped with an interchangeable commercial CdTe-based PCD [XC-Hydra FX50, Direct Conversion AB (Now with Varex Imaging)]. The PCD was operated under the charge sharing correction (CSC) mode with a measured dead time of 1.3 *μ*s, and two voltage thresholds were used to suppress electronic noise and to split the acquired photon counts into high-energy (HE) and total-energy (TE) photon count bins.

The x-ray source in the imaging benchtop system is the same as that used in the electronic prototype testing. The tube was operated at 70 kVp under the radiographic mode, and the tube current was adjusted from 0.5 to 200 mA to obtain different x-ray flux levels at the detector surface: note that the 100 *μ*m pixels in the PCD used for these imaging studies are much smaller than the 4 mm diode pixel used for the prototype electronics, and so much more clinically relevant mA levels could be used in the imaging studies while keeping the counts per second per pixel at a reasonable level; there also are differences in the dead time between the two detectors and respective electronics determined using the paralyzable model (4.7 *μ*s for the 4 mm pixel vs 1.3 *μ*s for the commercial 100 *μ*m pixel array). In comparison to the experiments described in section 2.2.1 with the single CdTe pixel, we did not have the ability to modify the existing electronics in this PCD. Therefore, the outputs of the proposed charge summing circuitry, namely the total charge accumulated over the time window (*T*), were emulated using a CsI(Tl)/a-Si(H)-based flat panel EID (4030CB, Varex Imaging, USA). The pixel pitch of the EID (194 *μ*m) is approximately two times that of the PCD (100 *μ*m), which allowed us to emulate the 2 × 2 pixel block charge integration scheme. At each mA level, first, the PCD was irradiated and counts were recorded, then the PCD was replaced with the EID and irradiated under identical conditions.

**Figure 6.**
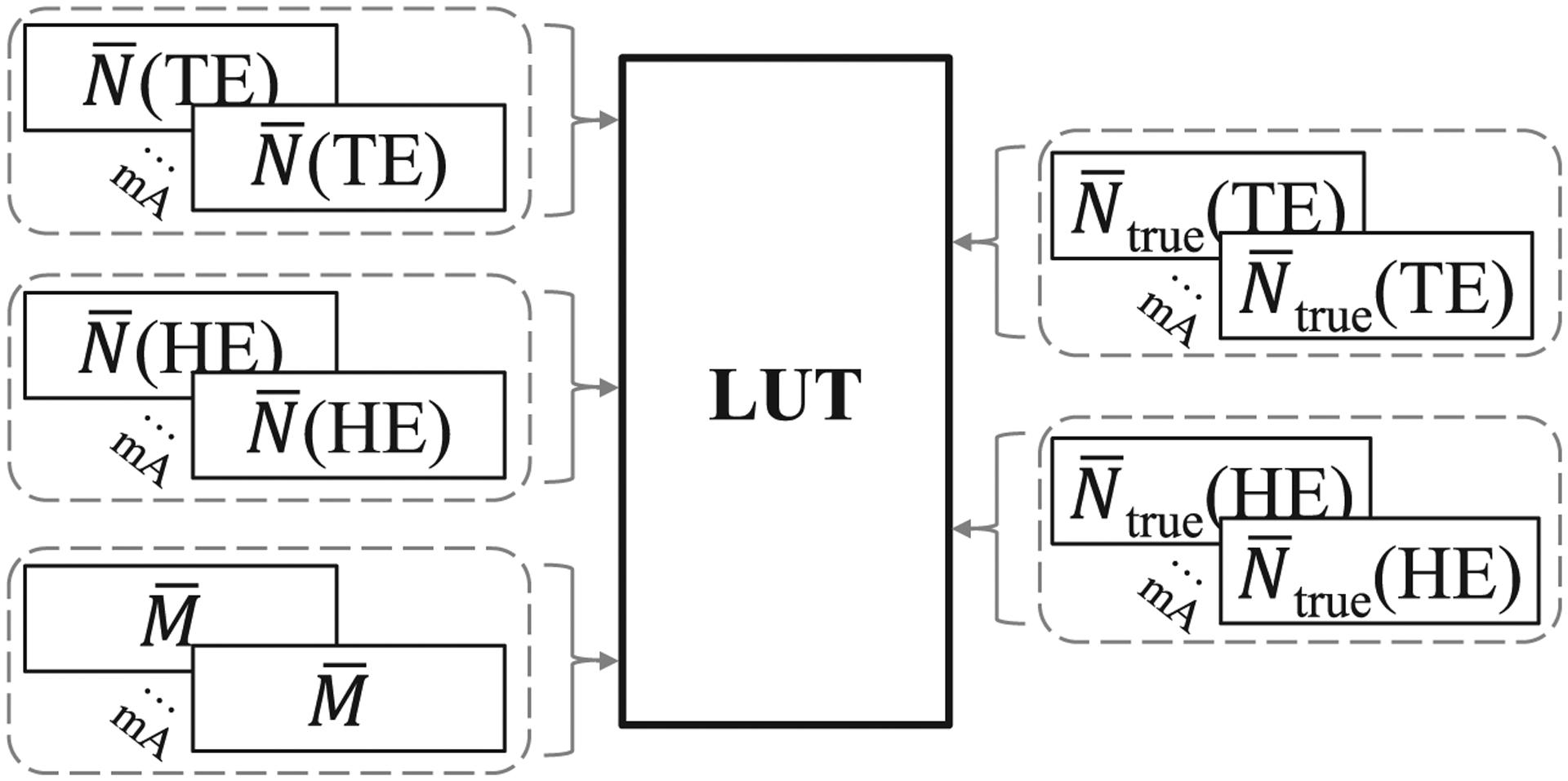
Diagram of the calibration procedure to generate the LUT for correcting photon counts. Inputs to generate the LUT are the mean measured values $\bar{N}(\text{TE})$, $\bar{N}(\text{HE})$, and $\bar{M}$ established through calibration across a range of mA levels, as well as ${\bar{N}}_{\text{true }}(\text{TE})$ and ${\bar{N}}_{\text{true }}(\text{HE})$ values estimated by linear extrapolation of $\bar{N}$ measured at the lowest mA levels where expected pile-up count loss is less than 1%.

**Figure 7.**
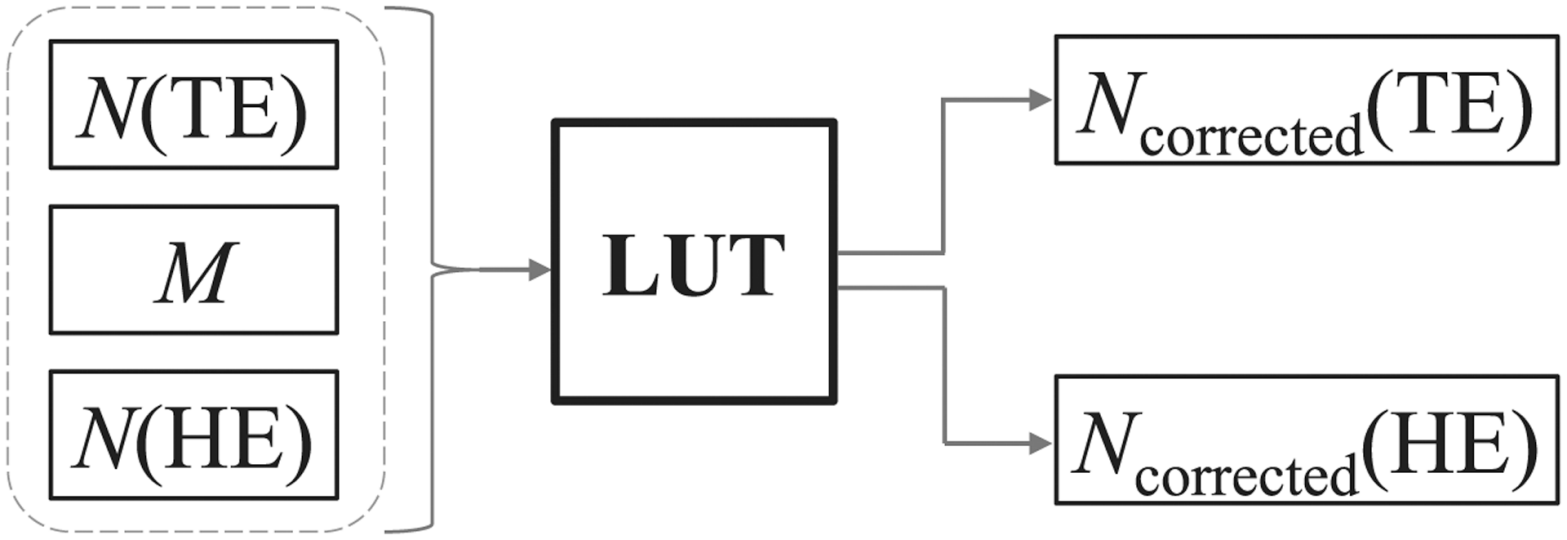
Flowchart of how the calibrated LUT is used in practice to correct for pile-up-induced count loss. The LUTs input is a vector containing single samples of *N*(TE), *N*(HE), and *M*, and its outputs are *N*_corrected_(TE) and *N*_corrected_(HE).

To establish calibration data for the count correction LUT, measured counts of the PCD for both the TE and HE bins and the digital outputs of the EID (*M*) were repeatedly measured in 50 air scan imaging frames for a given x-ray spectrum at each mA (flux) level; mean values $\bar{N}(\text{TE})$, $\bar{N}(\text{HE})$, and $\bar{M}$ were then calculated for each mA by averaging over the 50 measurement frames to reduce statistical uncertainty. The desired mean linear PCD counts $\left({\bar{N}}_{\text{true }}\right)$for each energy bin were estimated by performing linear extrapolation of $\bar{N}$ measured at the five lowest mA levels where the expected pile-up count loss is less than 1%. The count correction LUT is then generated for a given x-ray spectrum by taking the mean measured values $\bar{N}(\text{TE})$, $\bar{N}(\text{HE})$, and $\bar{M}$ established through calibration across a range of mA levels, and also inputting ${\bar{N}}_{\text{true }}(\text{TE})$ and ${\bar{N}}_{\text{true }}(\text{HE})$ values for each mA estimated by the extrapolation (figure 6). These two ${\bar{N}}_{\text{true }}$ values are then interpolated on a 3D grid of different combinations of $\left[\bar{N}(\text{TE}),\bar{N}(\text{HE}),\bar{M}\right]$ to generate the LUT across the range of possible combinations of these three parameters established by the calibration data. When calling the calibrated LUT to correct PCD images, its inputs are single samples of *N*(TE), *N*(HE), and *M*, since single samples are what’s available in clinical practice; the LUT takes these inputs and returns *N*_corrected_(TE) and *N*_corrected_(HE), as shown in figure 7, which are estimates of *N*_true_(TE) and *N*_true_(HE). Since not all possible combinations of *N*(TE), *N*(HE), and *M* occur in the calibration data of air scans, for example, due to beam hardening affecting the measured data, the measured input vector [*N*(TE), *N*(HE), *M*] is projected onto the point within the 3D grid of calibration data that is most consistent with the measured input vector, i.e. the closest calibration point in 3D space: the output values of *N*_corrected_(TE) and *N*_corrected_(HE) corresponding to this calibration point are then returned by the LUT. To evaluate the correction performance, we (1) plotted the uncorrected and corrected photon counts against mA to examine the linearity and accuracy; (2) compared radiological path lengths of PMMA calculated from uncorrected and corrected counts via log-normalization; (3) compared uncorrected and corrected log-normalized projections of a spatial resolution test pattern to determine how the larger EID pixels (emulating 2 × 2 pixel charge summing blocks) used to correct the measured photon counts may impact the spatial resolution of images formed using the smaller PCD pixels. For the log-normalization,(equation 2) was used to calculate ${\bar{N}}_{0}(\text{mA})$ where ${\bar{N}}_{0}\left({\text{mA}}_{ref}\right)$ was measured at 0.5 mA so that pulse pile-up was only significant in the measured *N* value.

## 3. Experimental results

### 3.1. Electronics prototype

**Figure 8.**
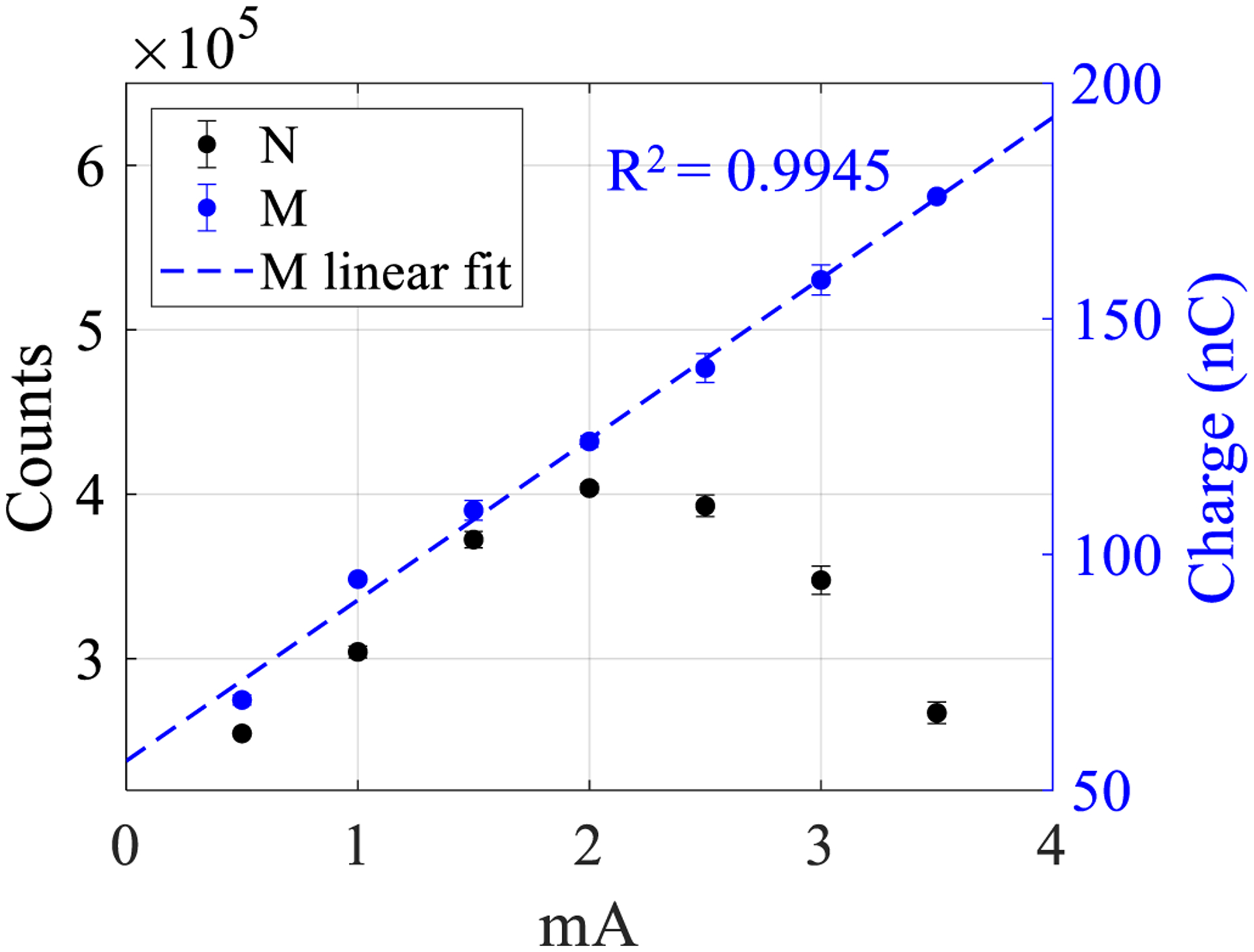
Plots of PCD counts *N* and simultaneously-measured time-integrated charge *M* with the prototype electronics over a 5 s time window for each mA. Error bars represent one standard deviation across repeated measurements.

Figure 8 demonstrates the ability of the proposed electronics to simultaneously measure recorded counts and time-integrated charge: despite the number of recorded counts being subject to pulse pile-up and exhibiting paralyzable behavior as the mA increases, the time-integrated charge from the same electric pulses does not suffer from pile-up and is linear with mA. The measurements are also reproducible, demonstrated by the error bars in figure 8. These results also confirm that the semi-Gaussian pulse area integrated by the charge summing channel is proportional to the total energy absorbed in the detector as x-ray flux changes with mA.

### 3.2. Pile-up correction experiments

**Figure 9.**
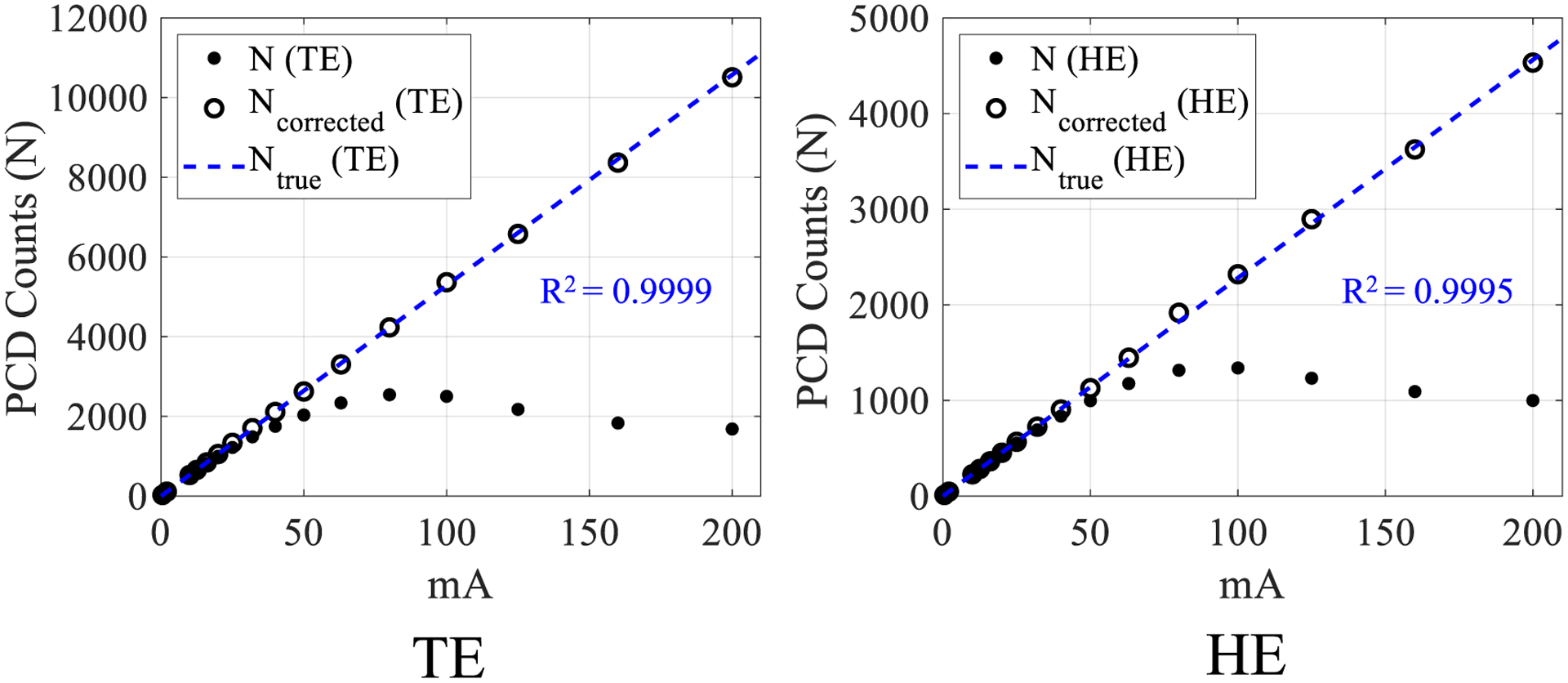
Plots of counts from the commercial PCD versus mA before and after the proposed correction for the total-energy (TE) bin and high-energy (HE) bin.

**Figure 10.**
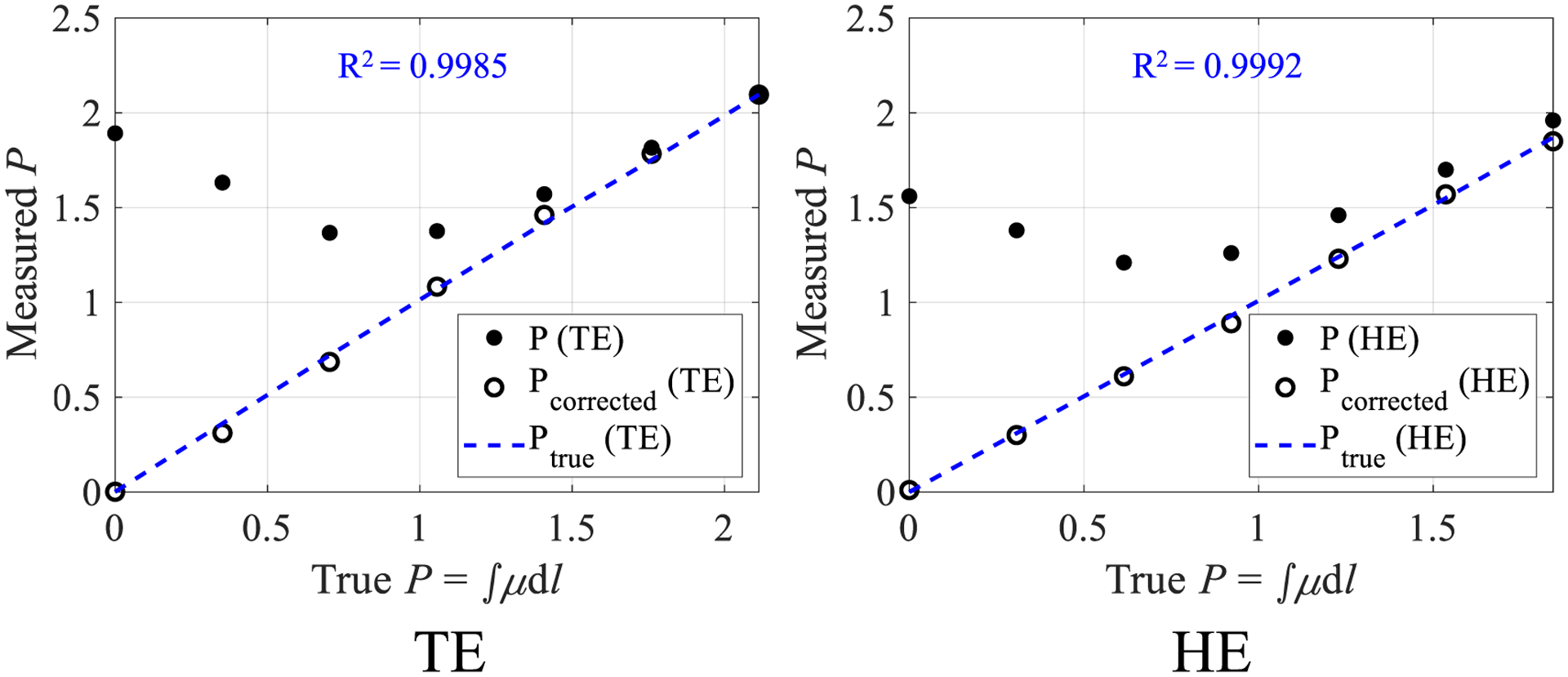
Plots of log-normalized projection (*P*) versus true radiological path length both before and after the proposed correction for the total-energy (TE) bin and high-energy (HE) bin. The data were collected at 200 mA from the commercial PCD.

**Figure 11.**
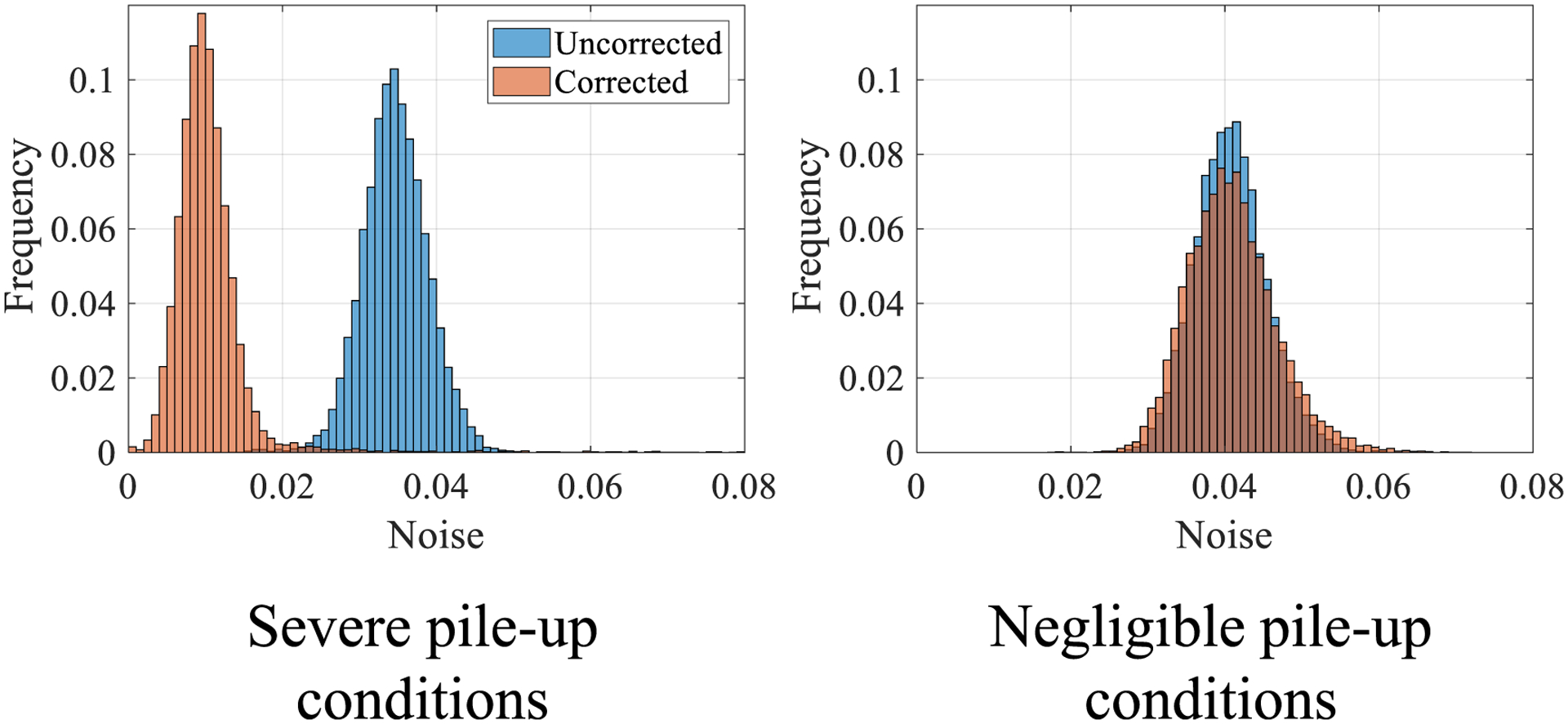
Left: frequency histograms of the noise measured in the total-energy bin of the commercial PCD for individual pixels across image frames demonstrating a difference in noise between the uncorrected and corrected images under severe pile-up conditions (*∫μ*d*l* = 0). Right: under negligible pile-up conditions (*∫μ*d*l* = 2.1), noise levels of uncorrected and corrected images are comparable. Step size of the horizontal axis: 0.001 [counts].

Figure 9 shows detected PCD counts as a function of mA without and with the proposed LUT correction. After correction, the PCD counts are linear with flux level and agree with the *N*_true_ values estimated via a linear extrapolation of the low mA data points for both the TE and HE bins. The proposed correction also does not alter the PCD counts measured at low flux levels where pulse pile-up is negligible. Another interesting result is that the peak value of the recorded PCD counts *N* occurs at a higher mA level (100 mA) in the HE bin compared to the TE bin (80 mA). This is likely due to increased pulse height and recorded energy from pile-up, pushing more photon counts to the high-energy bin as flux increases. Figure 10 shows log-normalized projection data as a function of the actual radiological path length of PMMA: As the material thickness (path length) decreases, pulse pile-up-induced count losses become more severe, and the measured path length artificially increases. After the proposed correction, the measured path lengths agree with the true values. The image noise was repeatedly measured in the TE bin log-normalized scans of air (severe pile-up) as well as the thickest PMMA condition in figure 10 with *∫μ*d*l* = 2.1 (negligible pile-up) at 200 mA: Histograms showing the distribution of noise across different image frames for individual detector pixels throughout the detector are shown in figure 11 for these two radiological path lengths. Under the severe pileup-up condition (*∫μ*d*l* = 0), the noise of the corrected image is lower than the uncorrected image. As a sanity check, when there is negligible pile-up, the noise levels of uncorrected and corrected images are comparable. This result suggests that when information is lost in the PCD due to pulse pile-up, the LUT-based correction may be able to exploit the information from the added charge integration measurement to reduce noise in the corrected image. However, when information is not lost due to pulse pile-up, i.e. under negligible pile-up conditions, the LUT does not seem to exploit any added information from the additional charge integration measurement to reduce image noise.

**Figure 12.**
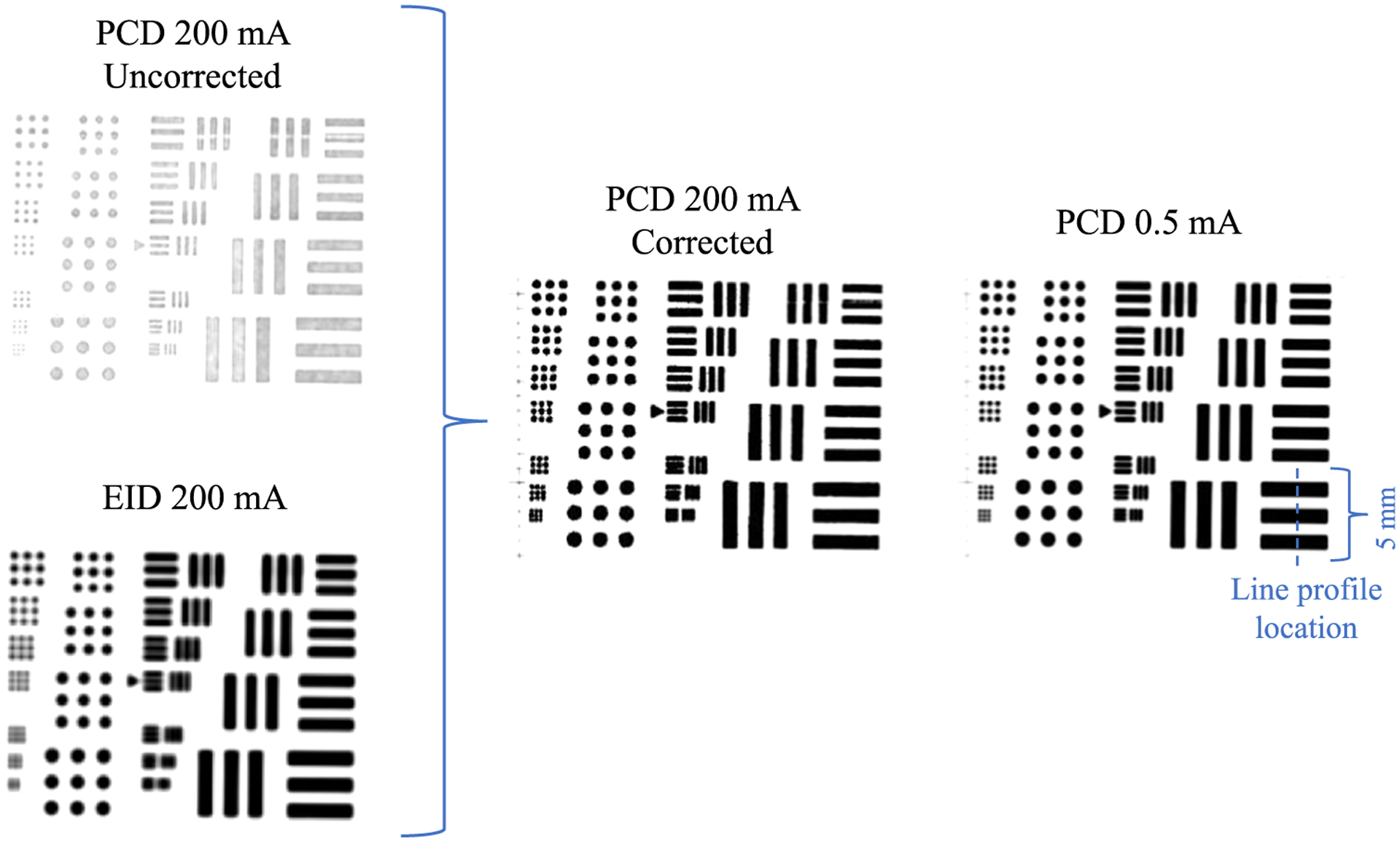
Log-normalized images of the spatial resolution test pattern demonstrating how the uncorrected PCD and EID data are combined to return a corrected PCD image. A PCD image with negligible pile-up (0.5 mA) is also shown for comparison. Window/level as well as total x-ray exposure are matched for all images.

**Figure 13.**
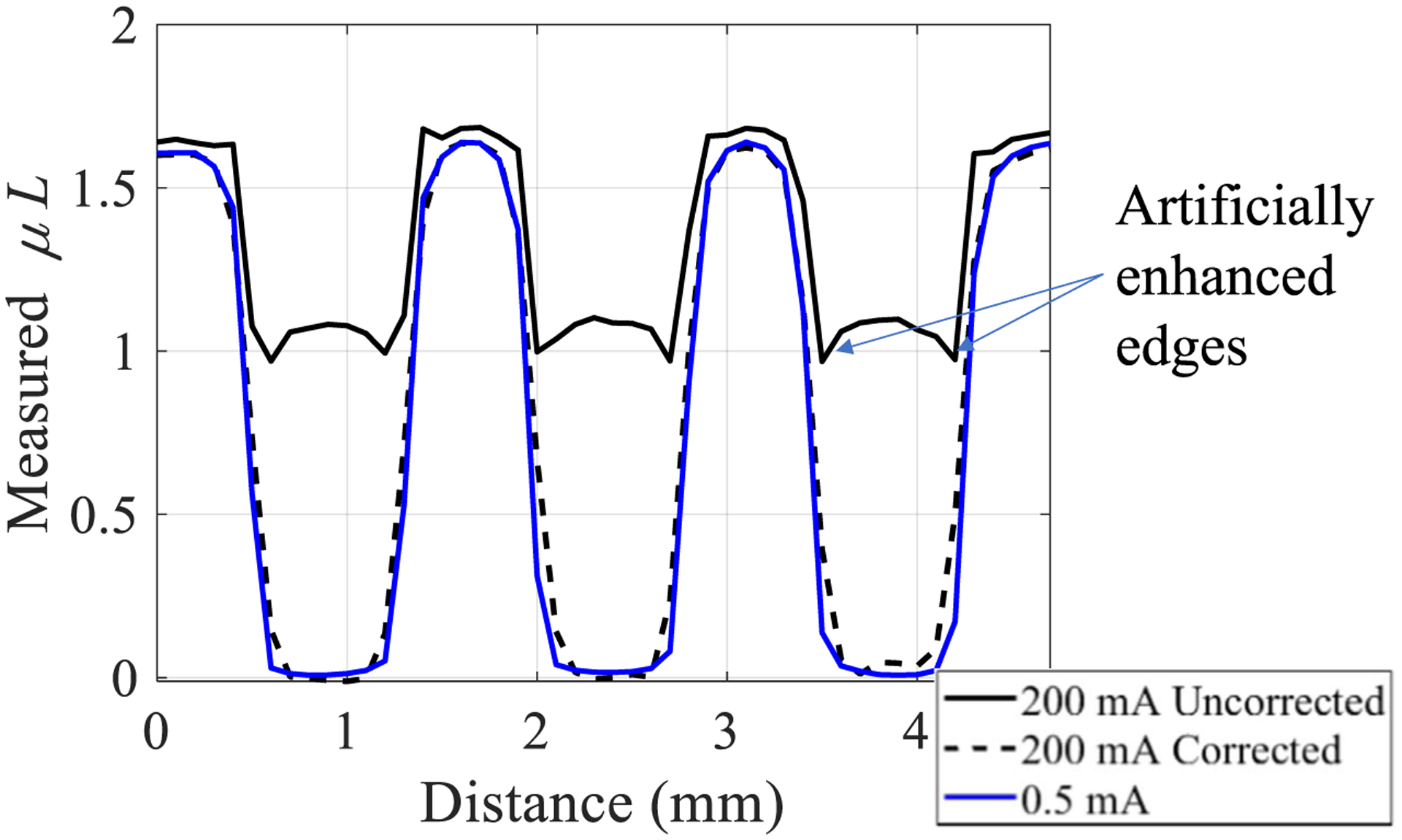
Line profiles through spatial resolution bar patterns. The measurement location is indicated in figure 12. Artificially enhanced edges are present in the uncorrected profile and removed after the correction is applied.

Log-normalized x-ray projection images of a spatial resolution test pattern are shown in figure 12, both before and after the proposed correction under severe pile-up (200 mA). The EID image used for correction is also shown along with the PCD image acquired with negligible pile-up at 0.5 mA for comparison. The correction restores the quantitative accuracy of the pile-up-free image without significantly degrading spatial resolution compared to the pile-up-free PCD image, despite the worse spatial resolution from the larger pixel size of the EID which emulates charge-summing electronics for every 2 × 2 PCD pixel block. This result demonstrates that: (1) the corrected image does in fact utilize the photon counting data and is not just based on the energy integrating data, and (2) the use of a larger 2 × 2 pixel block for integrating charge may not cause errors in the correction process. Line profiles of the lowest frequency horizontal bar patterns in the spatial resolution test pattern are shown in figure 13 and again demonstrate quantitative accuracy restoration with no spatial resolution loss due to the correction. Another noteworthy result is that artificially enhanced edges which are jointly caused by high-flux radiation and inter-pixel communication in the detector (Ji *et al* 2021) are removed after the proposed correction is applied. Note that these enhanced edges are only expected when the PCD is operated in the CSC mode (Ji *et al* 2021).

## 4. Discussion

Previous pile-up reduction methods include reducing the pulse shaping time or the pixel size, or on other hardware aspects of the imaging system, e.g. bowtie filters or fluence-field modulation. For a given set of system hardware and pulse pile-up condition, software-based methods show promise to correct for pulse pile-up, but typically rely on accurate detector modeling. However, how to address the degeneracy problem for paralyzable PCDs remains elusive when the only information available is the recorded counts. The novelty of this work lies in the utilization of the freely available information about the time-integrated charge which is linearly related to the actual input photon number for a given spectrum. By utilizing the existing charge integrator or by adding an integrator, the time-integrated charge (otherwise discarded) can supplement the pulse-mode counts for a better estimate of the true photon numbers. Experimentally, we demonstrated that the integrated charge can be used to correct for count losses in not only the total energy bin but also the high energy bin. As a side note, if the x-ray flux is pushed orders of magnitude higher than that presented in this work, the integrated charge will become nonlinear with mA due to accumulation of space charge in the CdTe sensor, reducing the electric field strength and increasing recombination losses; however, the fluxes typical in medical x-ray imaging are well below this point, and so nonlinearities in the integrated charge are not expected.

The idea of combining both charge integration and single photon counting in a detector pixel is not new, and in fact a prototype chip to do just that was tested by Kraft *et al* (2007), and was later extended to an 8 × 8 pixel matrix (Krüger *et al* 2008) and tested with several different Si, CdTe, and CdZnTe sensors (Fink *et al* 2009). These prototypes were tested in part under the motivation that ‘photon counting devices give excellent results for low to medium x-ray fluxes but saturate at high rates, while charge integration allows the detection of very high fluxes but is limited at low rates by the finite signal to noise ratio (Krüger *et al* 2008). In other words: at relatively low flux rates, i.e. in the low dose regime for medical x-ray imaging, electronic noise has a more pronounced impact on the signal-to-noise ratio for charge (energy) integrating detectors, but electronic noise does not impact the performance of PCDs; therefore PCDs should be used in the low dose regime. On the other hand, at relatively high flux rates, PCDs suffer from pulse pile-up whereas charge-integrating devices do not, and so charge integrating should be used in the high flux/high dose regime. Therefore, these previous works demonstrated that either the photon counting mode *or* the charge integrating mode can be used depending on x-ray the flux (although below the pile-up regime, both integrated charge and photon counts can be used simultaneously to estimate the mean energy of the detected x-ray spectrum). The novelty of the pulse pile-up correction method presented in this work is that the charge integrating device is never used directly in image formation, and is always used to correct the photon counting readout for pulse pile-up. Therefore, this work has a similar goal to the work presented by Kraft *et al* in that the simultaneous photon counting and charge integrating device is used to extend the dynamic range of the x-ray detector, but the difference is that our proposed method still uses the photon counting data for image formation even under severe pile-up conditions, and so the benefits of PCDs such as energy-resolving capabilities for material decomposition are retained even under high x-ray flux when paralyzable behavior is present.

**Figure 14.**
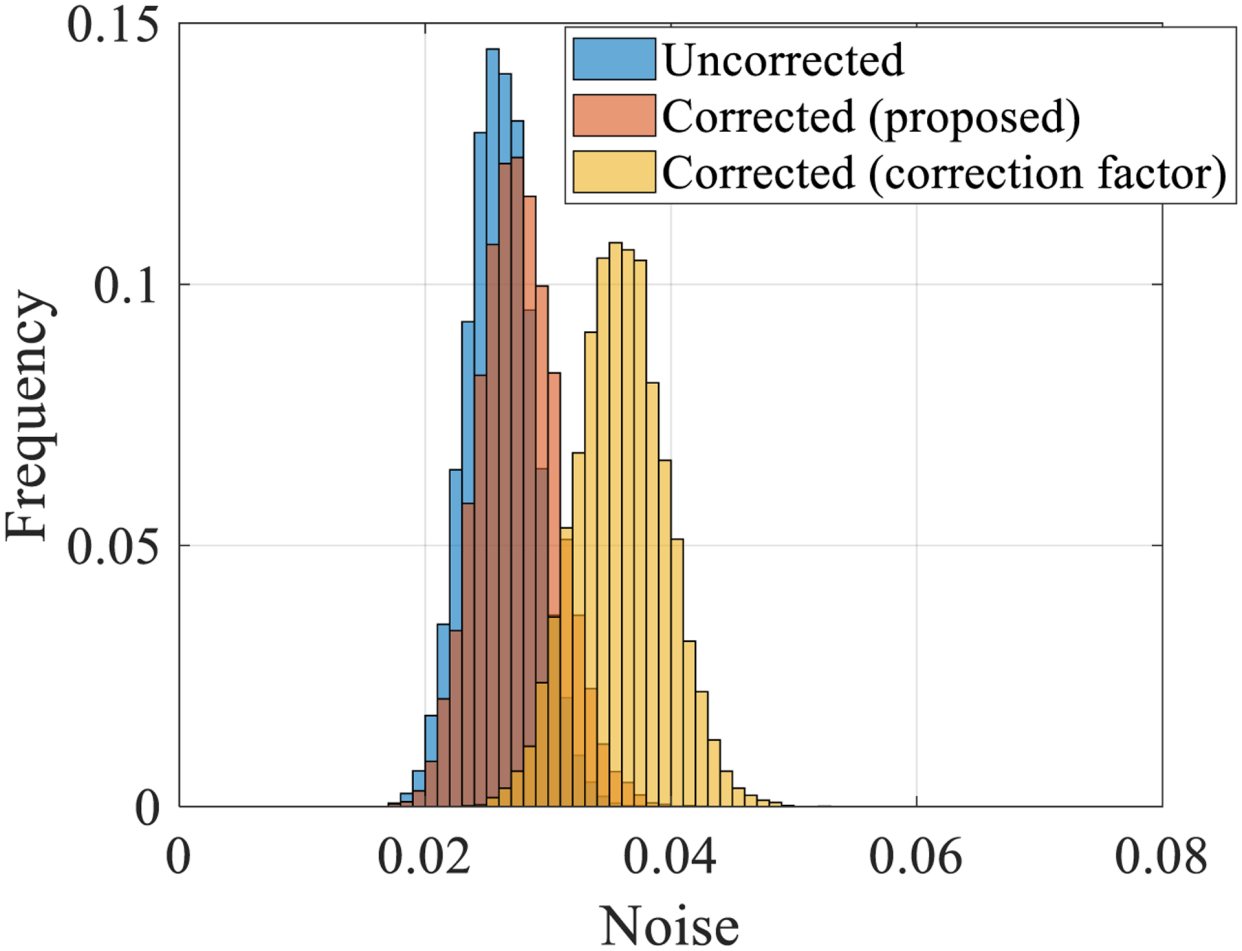
Frequency histograms of the noise measured in the total-energy bin for individual detector pixels across image frames demonstrating that the simple correction method of scaling the measured counts by a correction factor resulted in increased noise compared to the proposed correction method (*∫μ*d*l* = 1.7). Step size of the horizontal axis: 0.001 [counts].

Another major novelty with the proposed integrated charge-based correction method is that it is applicable to both paralyzable and non-paralyzable PCDs. However, for non-paralyzable PCDs as well as paralyzable PCDs that are operated at relatively low flux such that the measured count rate monotonically increases with the true count rate, pulse pile-up-induced count loss can be corrected for by simply multiplying each recorded counts measurement with a correction factor greater than 1 so that the expected value of the recorded counts will be proportional to the true counts (Cohen 1974). A limitation to this traditional method is that, unlike the proposed method, it is not applicable for paralyzable PCDs operated under high x-ray flux, due to one value of recorded count rate corresponding to two values of true count rate. Additionally, even under relatively low flux where the paralyzable nature of the PCD is not an issue and the simple correction factor method can be applied, this simple correction factor method increased noise in our measurements after log-normalization, as shown in figure 14. Therefore, the proposed pile-up correction method may have a noise benefit over this traditional correction method. However, a limitation of this work related to the noise analysis is that for the experimental studies using the commercial PCD array, the proposed parallel charge summing channel was emulated using separately acquired and spatially registered EID data. In this case, rather than the same pulses being sent to both the counter and the charge integrator, separate acquisitions are done for the photon counting and charge integrating that have uncorrelated noise properties, which can lead to different statistical behavior of the corrected counts after passing through the LUT. Therefore, the actual noise benefit of the proposed simultaneous counting and integrating measurements may be slightly different from the noise benefits presented in figure 11.

In addition to further evaluation of the noise properties of the proposed simultaneous counting and integrating, future work will involve implementing the proposed correction method for CT imaging studies rather than solely looking at x-ray projections. CT images can reveal subtle contrast differences that may not be easily visible in projection images, and so this would likely be a more rigorous test of the proposed correction scheme. Future work should also involve imaging a multitude of different materials in addition to air and PMMA, e.g. iodine, calcium, etc to ensure that the proposed LUT-based correction does not corrupt the measured spectral information from the PCD when higher-*Z* materials are imaged, since these materials will more aggressively harden the x-ray beam and can push the measured *N*(TE), *N*(HE), and *M* values further away from the calibration data acquired with air scans.

In addition, since the charge-summing nodes in PCDs can be used for correction of charge sharing between detector pixels, it is important to validate that collecting the charge from these nodes for the proposed pile-up correction does not impact the ability of charge-summing nodes to reconstruct shared charge across pixels: If either of these uses of the charge stored in these nodes is impacted by the other, then the operator may have to choose between the pile-up correction or charge sharing correction, which is undesirable. Lastly, in this work our commercial PCD array was operated under the CSC mode, which has the longest dead time of any mode for this PCD and was measured to be 1.3 *μ*s: this was done to test the proposed pulse pile-up correction under the most difficult conditions for this detector. The dead time of 1.3 *μ*s is within the range of other PCD models (Taguchi and Iwanczyk 2013), which are estimated from their reported maximum count rates to be around 3.0 *μ*s down to around 30 ns. Although pulse pile-up is less of an issue for PCDs with shorter dead times, there exists a trade-off between dead time and energy resolution, especially when the longer dead time is related to an added charge sharing correction (Treb *et al* 2021). Therefore, it may not always be desirable to use PCDs with the shortest available dead time.

## 5. Conclusions

In PCDs, the time-integrated charge accumulated over the period of each image frame can be collected in parallel with digital counting to correct for pile-up-induced count losses, even in paralyzable PCDs. This work provides the first experimental demonstration of the feasibility of (1) simultaneous photon counting and charge integration, and (2) correcting pile-up-induced count losses for two energy bins by utilizing the integrated charge. The proposed lookup table-based method can accurately estimate radiological path lengths from pile-up-distorted PCD counts in multiple energy bins using additional integrated charge data without sacrificing spatial resolution, and may have noise benefits over traditional methods that use a simple scaling factor for pile-up correction.

## Data Availability

The data cannot be made publicly available upon publication because the cost of preparing, depositing and hosting the data would be prohibitive within the terms of this research project. The data that support the findings of this study are available upon reasonable request from the authors.
